# Prevalence of symptoms, ever having received a diagnosis and treatment of depression and anxiety, and associations with health service use amongst the general population in two Russian cities

**DOI:** 10.1186/s12888-020-02938-w

**Published:** 2020-11-12

**Authors:** Sarah Cook, Alexander V. Kudryavtsev, Natalia Bobrova, Lyudmila Saburova, Diana Denisova, Sofia Malyutina, Glyn Lewis, David A. Leon

**Affiliations:** 1 Department of Community Medicine, UiT the Arctic University of Norway, Tromsø, Norway; 2 Faculty of Epidemiology and Population Health, London School of Hygiene & Tropical Medicine, London, UK; 3 Northern State Medical University, Arkhangelsk, Russian Federation; 4 Institute of Philosophy and Law, Ural Branch of the Russian Academy of Sciences, Ekaterinburg, Russian Federation; 5 Research Institute of Internal and Preventive Medicine, Branch of Institute of Cytology and Genetics, Siberian Branch of the Russian Academy of Sciences, Novosibirsk, Russian Federation; 6 Novosibirsk State Medical University, Russian Ministry of Health, Novosibirsk, Russian Federation; 7 Division of Psychiatry, University College London, London, UK; 8 International Laboratory for Population and Health, National Research University Higher School of Economics, Moscow, Russian Federation

**Keywords:** Russian Federation, Depression, Anxiety, Mental disorders, Treatment of mental disorders, Anti-depressants, Anxiolytics

## Abstract

**Background:**

Little is known about the burden of common mental disorders in Russia despite high levels of suicide and alcohol-related mortality. Here we investigated levels of symptoms, self-reports of ever having received a diagnosis and treatment of anxiety and depression in two Russian cities.

**Methods:**

The study population was men and women aged 35–69 years old participating in cross-sectional population-based studies in the cities of Arkhangelsk and Novosibirsk (2015–18). Participants completed an interview which included the PHQ-9 and GAD-7 scales, questions on whether participants had ever received a diagnosis of depression or anxiety, and health service use in the past year. Participants also reported current medication use and medications were coded in line with the WHO anatomical therapeutic classification (ATC). Depression was defined as PHQ-9 ≥ 10 and Anxiety as GAD-7 ≥ 10.

**Results:**

Age-standardised prevalence of PHQ-9 ≥ 10 was 10.7% in women and 5.4% in men (GAD-7 ≥ 10 6.2% in women; 3.0% in men). Among those with PHQ-9 ≥ 10 17% reported ever having been diagnosed with depression (equivalent finding for anxiety 29%). Only 1.5% of those with PHQ-9 ≥ 10 reported using anti-depressants and 0.6% of those with GAD-7 ≥ 10 reported using anxiolytics. No men with PHQ-9 ≥ 10 and/or GAD-7 ≥ 10 reported use of anti-depressants or anxiolytics. Use of health services increased with increasing severity of both depression and anxiety.

**Conclusion:**

There was a large gap between symptoms and reporting of past diagnosis and treatment of common mental disorders in two Russian cities. Interventions aimed at improving mental health literacy and reducing stigma could be of benefit in closing this substantial treatment gap.

**Supplementary Information:**

The online version contains supplementary material available at 10.1186/s12888-020-02938-w.

## Background

Common mental disorders (depression and anxiety) are important public health concerns worldwide [1, 2]. Relatively little attention has been given to the burden of common mental disorders and their treatment in Russia, despite the country having high rates of suicide and alcohol-related mortality [3–6]. Depressive symptoms have been shown to predict cardiovascular and all-cause mortality in the Russian population [7].

Three population-based studies from the start of the millennium found a high prevalence of symptoms of depression [8–10] and anxiety [9]. The HAPIEE study which assessed depression using the Centre for Epidemiologic studies depression scale (CES-D) > 16 among men and women aged 45–64 in the city of Novosibirsk (1999–2000) found a prevalence of 23% in men and 44% in women [8]. The Arkhangelsk Study (2000) assessed depression and anxiety using binary questions about feeling depressed in the past 2 weeks/anxious in the past year found prevalence of depression 10% in men and 34% in women and of anxiety 25% in men and 53.2% in women [9]. A study of 885 adults aged 18–64 living in rural Udmurtia in 1995 found 27.3% participants met ICD-10 criteria for a mood disorder in the past month when interviewed using the Composite International Diagnostic Interview [10]. In the past 20 years, Russia has undergone substantial political, economic and social change [11]. It is therefore timely to investigate the burden of common mental disorders and associated levels of treatment in present day Russia. Early findings from the more recent ESSE-RF multi-centre study (2013–14) suggests the burden remains high with a 25.6% prevalence of depression and 46.3% prevalence of anxiety among 16,877 men and women aged 25–64 years old assessed using the Hospital Anxiety and Depression Scale (HADS) score ≥ 8 [12]. None of these studies investigated issues to do with levels of diagnosis or treatment within the general population. However a study of 155 primary care users from St Petersburg in Russia has found low levels of treatment for depression among 55 participants identified as having symptoms using CES-D, particularly with pharmacotherapy with no participants with depression using antidepressants [13].

Treatment for mental health problems in Russia primarily takes place within specialist services with a focus on treatment delivery through psychiatric outpatient dispensaries and hospitals [14–17]. Seeking treatment privately is also an option for those who can afford it. Assessment and treatment within primary care is rare [15, 16, 18, 19]. However if those with depression and anxiety are more likely to attend general health care services this may equate with missed opportunities for care. One potential reason for low levels of diagnosis and treatment is lack of contact with health services in general however in several populations, including the primary care users in the study from St Petersburg [20], common mental disorders have been found to be associated with higher use of general health services [20–22].

In this study we investigated the prevalence of symptoms depression and anxiety, self-reports of ever being diagnosed with depression and anxiety, and one specific aspect of treatment (use of anti-depressants and anxiolytic medication) in the general population in two Russian cities. We also investigated the associations between severity of symptoms and health service use. Since physical co-morbidities may drive an association between mental health symptoms and health care use findings were stratified by the presence or absence of chronic physical health problems.

## Methods

### Study population

The study population was men and women aged 35–69 years participating in the Know Your Heart study (2015–18). This was a cross-sectional population-based survey set up to investigate reasons for high cardiovascular disease mortality in Russia that collected a wide-range of information about participants’ physical and mental health and health service use and medications. Detailed methodology of the study including response rates have been described previously [23].

Recruitment took place in two Russian cities – Arkhangelsk, in the North of European Russia and Novosibirsk in Western Siberia. Novosibirsk is the third largest city in Russia, after Moscow and St Petersburg, with a population of 1,500,000 while Arkhangelsk is a smaller city with a population of approximately 350,000 people. Data from the Russian census 2010 shows that the age distribution of the two cities was similar to the National average but the proportion of people with higher education was higher in Novosibirsk compared to the Urban Russian population as a whole while in Arkhangelsk it was similar. Consistent with patterns for all-cause mortality and cardiovascular disease mortality at the time of study Russian state statistics showed higher mortality from both suicide and alcohol-related poisoning in Arkhangelsk than Novosibirsk.

Addresses of eligible participants were identified from Territorial Health Insurance Fund records with sampling stratified by age, sex and district (four districts per city). Trained interviewers visited randomly selected addresses and invited participants of the correct expected age and sex to take part in the study. Excluding addresses which were invalid or where no one of the correct age or sex was resident response rates were 53.1% in Arkhangelsk and 26.5% in Novosibirsk. The target population of the study was men and women aged 35–69.

If participants agreed to take part the interviewer carried out a face to face interview, in the majority of cases in the participants’ home, which included the questions of the PHQ-9, GAD-7, whether participants had ever received a diagnosis of depression or anxiety, self-reported diseases, socio-demographic factors and health behaviours (smoking and alcohol use). After this interview participants were invited to attend a health check examination at a polyclinic which 89% of participants attended. Data on medication use were collected at the health check.

### Measurement of symptoms, ever having received a diagnosis and treatment of depression and anxiety

Depression was measured using the PHQ-9 [24]. This is a nine-item scale with questions on symptoms of depression in the past 2 weeks. Each question has four response options (not at all/several days/more than half of the days/nearly every day). A severity score was calculated by summing responses to each question with “not at all” responses scored as zero and “nearly every day” as three. A cut point of ≥10 or above was used to define moderate depression as a binary outcome and the following cut points to define depression severity: 5–9 mild depression, 10–14 moderate depression, 15–19 major depression and ≥ 20 major severe depression [24]. Anxiety was measured using the GAD-7 a 7-item scale with questions on symptoms of anxiety in the past 2 weeks [25]. Scoring and calculation of the severity score was the same as for the PHQ-9. A cut point of ≥10 or above was used to define moderate anxiety as a binary outcome and the following cut points to define anxiety severity: 5–9 mild anxiety, 10–14 moderate anxiety and ≥ 15 severe anxiety [25]. Standard Russian translations of the PHQ-9 and GAD-7 were used (www.phqscreeners.com).

Ever having received a diagnosis of depression or anxiety was assessed through self-report using these two questions: “Have you ever been told by a doctor (been diagnosed) that you have depression?” and “Have you ever been told by a doctor (been diagnosed) that you have anxiety?” with response categories yes/no.

Participants were asked to bring all their medications to the health check where medically trained interviewers asked about current medication use and recorded the name, dose, indication and frequency of use (up to 7 medications). If participants did not bring medications with them (73% of participants did not bring medications) they were asked verbally to report this information. Medications were coded using the International WHO Anatomical Therapeutic Chemical (ATC) classification system (https://www.whocc.no/). Use of anti-depressant medication was defined as any medication in class N06A and anxiolytic medication as any medication in class N05B. Use of drugs containing hypnotic or sleeping medications defined as any medication in class N05C were also considered.

### Health service use

Use of health services was measured by considering the number of visits to a doctor and the number of hospital admissions. Participants were asked how many times in the past 12 months they had visited the following types of physician: district physician/polyclinic cardiologist/other polyclinic specialist/hospital cardiologist/other hospital doctor. For each type of doctor visited the response options were integers 0 to 5+. The visits to each type of doctor were summed together treating “5+” as 5 allowing for a maximum possible number of 25 visits to a doctor. Number of hospital admissions was measured from the question “In the last 12 months, how many times have you been hospitalised (stayed in hospital overnight)?” The number of times was an open response (no categories given).

The number of medications used per participant among the sub-set of participants with data on medication use was also considered as a further indicator of medical care.

### Self-reported physical morbidity

Analyses on associations with health service and medication use were stratified by report of physical co-morbidities in the baseline interview “Have you ever been told by a doctor (been diagnosed) that you have: cancer, angina, stroke, rheumatoid arthritis, osteoarthritis, asthma, diabetes, chronic lung disease, myocardial infarction, kidney disease, heart failure?” This was to investigate to what extent associations may be due to poorer physical health leading to both worse mental health and increased use of health care.

### Other variables of interest

Potential confounders of the association between common mental disorders and health service use considered were demographic factors (age, sex and marital status), socio-economic factors (education (classified into three groups lower, middle and higher) self-perceived financial status measured on a 6 item likert scale and employment status (in regular paid employment or not)) and health behaviours (smoking status (current smoker, ex-smoker, never smoker), alcohol use measured in terms of volume of ethanol from beer, wine, and spirits in the past year and CAGE score [26] for problem drinking adapted to a 12 month reference period. While current socio-economic circumstances and health behaviours are also potentially mediators of the relationship between mental health and health service use our aim here was to investigate whether common mental disorders were associated with increased help seeking from medical services which may be confounded by worse physical health.

### Statistical analysis

Prevalences of symptoms indicating moderate depression and anxiety, ever having received a diagnosis and treatment in the general population sample were calculated stratified by sex and city and directly standardised by age to the European 2013 Standard Population for those aged 35–69. Between city differences in prevalence of symptoms were investigated by fitting logistic regression models with moderate depression as the outcome, city as the main exposure and adjustments for a) age and sex b) model 1 plus socio-economic factors (education, financial status, employment status) and c) model 2 plus health behaviours (smoking, alcohol use).

Distribution of use of health service variables were skewed therefore median (IQR) number of visits to a doctor, hospital admissions and use of medications were considered by category of depression and anxiety severity and modelled using negative binomial regression as an alternative to poisson regression due to over-dispersion of the outcome variables. Due to small number of participants with severe major depression (*n* = 27) the two major depression categories were collapsed for these analyses. Models were fitted stratified by reporting of physical co-morbidities. Negative binomial regression models were fitted for each outcome and mental health exposure separately adjusting a) for age sex and city and b) adjusting for age, sex, city, marital status, socio-economic factors and health behaviours.

Statistical analyses were done in Stata 15 [27].

## Results

The sample size was 5077 participants aged 35–69 years at the time of the baseline interview (42.8% male, mean age 54 (SD 9.8)). Of these participant 4060 attended the health check and had data available on use of medications.

### Prevalence of symptoms, ever having received a diagnosis and treatment of depression and anxiety

The age-standardised prevalence of moderate depression (PHQ-9 ≥ 10) for those aged 35–69 in Novosibirsk was 10.6% (95% CI 9.4, 11.9%) and in Arkhangelsk 6.3% (95% CI 5.4, 7.3%). The age-standardised prevalence of moderate anxiety (GAD-7 ≥ 10) was 6.0% (95% CI 5.1, 7.0%) in Novosibirsk and 3.8% (95% CI 3.0, 4.6%) in Arkhangelsk.

The odds of moderate depression were higher in Novosibirsk after adjusting for age and sex (OR 1.76 95% CI 1.43, 2.16). The difference between sites was attenuated but not fully explained by adjustment for socio-economic factors (OR 1.53 95% CI 1.24, 1.89). Additional adjustment for differences in health behaviours between the study populations in each city did not explain this further (1.53 95% CI 1.27, 1.93). Distribution of socio-demographic factors and health behaviours between the study populations of the two cities are shown in Supplementary Table 1.

The distribution of severity scores on the PHQ-9 and GAD-7, prevalence of scores≥10 and reports of ever having been diagnosed with depression and anxiety by sex and city are shown in Table 1. There were consistently higher prevalences in Novosibirsk compared to Arkhangelsk but the pattern of results was the same in both cities: prevalence of moderate depression (PHQ-9 ≥ 10) and anxiety (GAD-7 ≥ 10) was appreciably higher in women than in men. The prevalence of reporting ever having received a diagnosis of depression was substantially lower than point prevalence of moderate depression based on symptoms. This was a consistent finding in men and women. Levels of reporting of ever having received a diagnosis of anxiety were slightly higher than point prevalence in women and similar to point prevalence in men.

**Table 1:** Severity of depression and anxiety and age-adjusted prevalence of moderate depression and anxiety (symptoms and report of ever having received a diagnosis) by city and sex

	Arkhangelsk				Novosibirsk				Both sites			
	Men (*n* = 1022)		Women (*n* = 1430)		Men (*n* = 1151)		Women (*n* = 1474)		Men (*n* = 2173)		Women (*n* = 2904)	
	N	(%)	N	(%)	N	(%)	N	(%)	N	(%)	N	(%)
PHQ-9 severity score												
No symptom (< 5)	784	(76.7)	902	(63.1)	807	(70.1)	827	(56.1)	1591	(73.2)	1729	(59.5)
Mild depression (5–9)	198	(19.4)	407	(28.5)	259	(22.5)	444	(30.1)	457	(21.0)	851	(29.3)
Moderate depression (10–14)	29	(2.8)	87	(6.1)	56	(4.9)	138	(9.4)	85	(3.9)	225	(7.8)
Major depression (15–19)	9	(0.9)	27	(1.9)	24	(2.1)	52	(3.5)	33	(1.5)	79	(2.7)
Major depression- severe (≥ 20)	2	(0.2)	7	(0.5)	5	(0.4)	13	(0.9)	7	(0.3)	20	(0.7)
Age-standardised prevalence of moderate depression PHQ9 ≥ 10 (95% CI)	3.7	(2.7, 5.0)	8.1	(6.8, 9.6)	6.9	(5.5, 8.6)	13.3	(11.7, 15.2)	5.4	(4.5, 6.4)	10.7	(9.6, 11.9)
Age-standardised prevalence ever diagnosed with depression^a^ (95% CI) (missing = 11)	2.6	(1.8, 3.9)	6.7	(5.5, 8.2)	1.3	(0.1, 2.2)	3.5	(2.7, 4.5)	2.0	(1.5, 2.7)	5.1	(4.3, 5.9)
GAD-7 Severity score												
No symptoms (< 5)	888	(86.9)	1070	(74.8)	957	(83.2)	1053	(71.4)	1845	(84.9)	2123	(73.1)
Mild anxiety (5–9)	112	(11.0)	292	(20.4)	150	(13.0)	312	(21.2)	262	(12.1)	604	(20.8)
Moderate anxiety (10–14)	14	(1.4)	46	(3.2)	34	(3.0)	73	(5.0)	48	(2.2)	119	(4.1)
Severe anxiety (≥ 15)	8	(0.8)	22	(1.5)	10	(0.9)	36	(2.4)	18	(0.8)	58	(2.0)
Age-standardised prevalence of moderate anxiety GAD-7 ≥ 10 (95% CI)	3.8	(2.8, 5.2)	7.6	(6.3, 9.1)	2.2	(1.4, 3.3)	4.9	(3.8, 6.1)	3.0	(2.4, 3.9)	6.2	(5.4, 7.2)
Age-standardised prevalence ever diagnosed with anxiety^b^ (95% CI) (missing = 12)	4.1	(3.0, 5.6)	12.1	(10.5, 14.0)	2.0	(1.2, 3.0)	5.7	(4.6, 7.0)	3.0	(2.3, 3.9)	8.9	(7.9, 10.0)

^a^ 11 participants responded “difficult to answer”. They were coded as missing

^b^12 participants responded “difficult to answer”. They were coded as missing

Use of anti-depressants and anxiolytic medications in the general population was extremely low in both cities (10 participants reported use of anti-depressants and 8 reported use of anxiolytics data from both cities combined). Use of sleeping medications in the general population (*n* = 27 0.7%) was slightly more common than either anti-depressants (0.3%) or anxiolytics (0.2%) but also very low. The prevalence of use of medications among those with PHQ-9 ≥ 10 and GAD-7 ≥ 10 is shown in Table 2. Levels of treatment of depression and anxiety with anti-depressant and anxiolytic medication was extremely low and no men with symptoms of either condition were receiving any pharmacological treatment.

**Table 2:** Use of antidepressants, anxiolytics and sleeping medications among those with PHQ-9 ≥ 10 and GAD-7 ≥ 10

	N with medication data	Use of anti-depressants (N06A)	Use of anxiolytics (N05B)	Use of sleeping medication (N05C)
Moderate depression (PHQ-9 ≥ 10)				
**Total**	**334**	**1.5%**	**0.6%**	**1.5%**
*Men*	*86*	*0.0%*	*0.0%*	*0.0%*
*Women*	*248*	*2.0%*	*0.8%*	*2.0%*
Moderate anxiety (GAD-7 ≥ 10)				
**Total**	**181**	**2.2%**	**0.6%**	**2.8%**
*Men*	*46*	*0.0%*	*0.0%*	*0.0%*
*Women*	*138*	*2.9%*	*0.7%*	*3.7%*
Co-morbid moderate depression and anxiety (PHQ-9 & GAD-7 ≥ 10)				
**Total**	**121**	**3.3%**	**0.8%**	**3.3%**
*Men*	*26*	*0.0%*	*0.0%*	*0.0%*
*Women*	*95*	*4.2%*	*1.1%*	*4.2%*

**Fig. 1:**
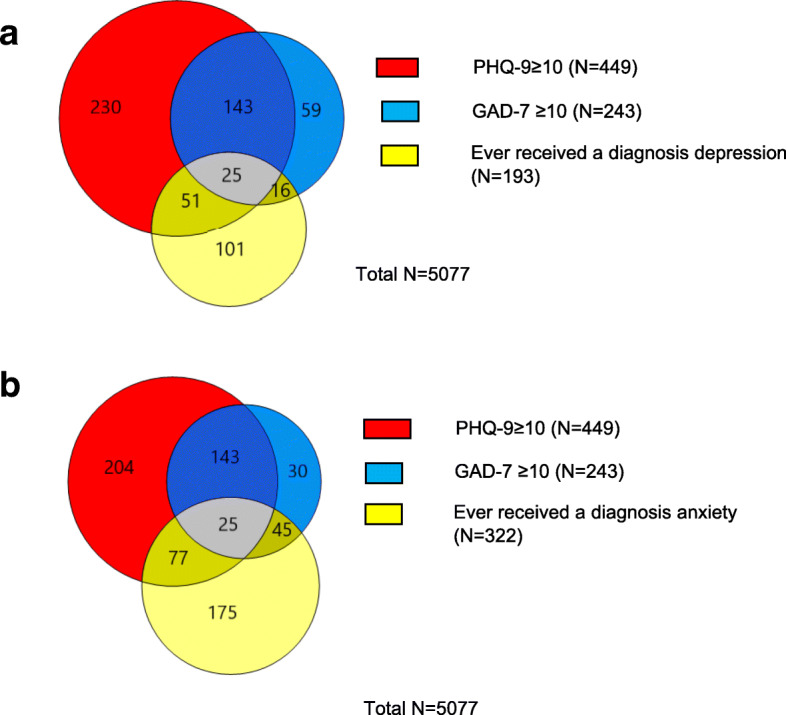
**a** Overlap in symptoms of depression, anxiety and report of ever receiving a diagnosis of depression. **b** Overlap in symptoms of anxiety, depression and report of ever receiving a diagnosis of anxiety

The overlap between PHQ-9 ≥ 10 and GAD-7 ≥ 10 and report of ever having received a diagnosis of depression is shown in Fig. 1a. The equivalent for reporting ever having received a diagnosis of anxiety is shown in Fig. 1b. Despite differences in absolute prevalences the findings for men and women were similar and the results are shown for men and women combined. The prevalence of co-morbidity was very high – particularly for those with GAD-7 ≥ 10 of whom 69.1% also had PHQ-9 ≥ 10 while 37.4% of those with PHQ-9 ≥ 10 also had GAD-7 ≥ 10. Among participants with PHQ-9 ≥ 10 the proportion who reported they had ever received a diagnosis of depression was low (16.9%). Prevalence of reporting ever receiving a diagnosis of anxiety among participants with GAD-7 ≥ 10 was slightly higher (28.8%).

### Associations with use of health services and medications

The age and sex adjusted associations between severity of depression and anxiety with use of health services and medications among those with no self-reported physical co-morbidities are shown in Table 3. There were strong associations between both severity of depression and anxiety and the health service use outcomes: visits to a doctor and admissions to hospital and also some evidence for a trend with number of medications used with rates increasing with severity of the mental health conditions. Findings remained with only slight attenuation of rate ratios on adjustment for socio-demographic factors and health behaviours (Table 3). The strong association between severity of depression and anxiety and the measures of health service use was also seen in the sub-group of participants who reported one or more physical co-morbidity (Supplementary Table 2).

**Table 3:** Associations between severity of depression and anxiety with use of health services and medications (no self-reported physical health problems)

PHQ-9 Severity score	Number of visits to doctor in past 12 months (missing = 5)				Number of hospital visits in past 12 months				Number of medications used			
	Median (IQR)	Median (IQR)^a^	Age, sex and city adjusted rate ratio^a^	Fully adjusted rate ratio^a^,^b^	Median (IQR)	Median (IQR)^a^	Age, sex and city adjusted rate ratio^a^	Fully adjusted rate ratio^a,b^	Median (IQR)	Median (IQR)^a^	Age, sex and city adjusted rate ratio^a^	Fully adjusted rate ratio^a,b^
No symptom (< 5)	2 (0–4)	1 (0–3)	1.00 (ref)	1.00 (ref)	0 (0–0)	0 (0–0)	1.00 (ref)	1.00 (ref)	1 (0–2)	0 (0–1)	1.00 (ref)	1.00 (ref)
Mild depression (5–9)	3 (1–7)	2 (0–3)	1.34 (1.14, 1.56)	1.31 (1.12, 1.53)	0 (0–0)	0 (0–0)	1.42 (0.95, 2.11)	1.47 (0.98, 2.21)	1 (0–3)	0 (0–1)	1.16 (0.95, 1.43)	1.18 (0.96, 1.46)
Moderate depression (10–14)	4 (1–8)	1 (0–5)	1.41 (0.98, 2.03)	1.43 (0.99, 2.07)	0 (0–1)	0 (0–0)	1.83 (0.80, 4.18)	1.96 (0.85, 4.54)	2 (0–3.3)	1 (0–2)	1.38 (0.86, 2.21)	1.42 (0.88, 2.30)
Major depression (≥ 15)	5 (1–10)	4.5 (1.25–6.75)	2.91 (1.66, 5.12)	2.70 (1.55, 4.71)	0 (0–1)	0 (0–1)	5.37 (1.86, 15.53)	5.30 (1.82, 15.42)	2 (1–3)	1 (0–2)	2.49 (0.96, 6.42)	2.16 (0.82, 5.70)
Test for trend			*p* < 0.001	*P* < 0.001			*P* = 0.001	*P* = 0.001			*P* = 0.02	*p* = 0.02
GAD-7 Anxiety severity score												
No symptoms (< 5)	2 (0–5)	1 (0–3)	1.00 (ref)	1.00 (ref)	0 (0–0)	0 (0–0)	1.00 (ref)	1.00 (ref)	1 (0–2)	0 (0–1)	1.00 (ref)	1.00 (ref)
Mild anxiety (5–9)	3 (1–7)	2 (0–4)	1.38 (1.15, 1.65)	1.32 (1.10, 1.59)	0 (0–0)	0 (0–0)	1.52 (0.97, 2.39)	1.47 (0.93, 2.33)	1 (0–3)	0 (0–1)	1.17 (0.93, 1.49)	1.15 (0.90. 1.46)
Moderate anxiety (10–14)	3 (0–8)	2 (0–5.25)	1.84 (1.20, 2.81)	1.79 (1.17, 2.73)	0 (0–0)	0 (0–0)	2.85 (1.17, 6.92)	2.81 (1.15, 6.84)	1 (0–3)	1 (0–2.5)	1.87 (1.09, 3.19)	1.74 (1.01, 2.98)
Severe anxiety (≥ 15)	4 (1–8)	2.5 (0–5.75)	1.50 (0.70, 3.20)	1.53 (0.72, 3.27)	0 (0–1)	0 (0–0)	3.18 (0.69, 14.70)	2.54 (0.55, 11.76)	2 (0–4)	0 (0–3.5)	2.59 (0.75, 8.96)	3.13 (0.93, 10.57)
Test for trend			*p* < 0.001	*P* < 0.001			*P* = 0.002	*p* = 0.005			*P* = 0.005	*P* = 0.009

^a^Restricted to those who did not report diagnosis of cancer, angina, stroke, rheumatoid arthritis, osteoarthritis, asthma, diabetes, chronic lung disease, myocardial infarction, kidney disease or heart failure and no missing data on covariates (*n* = 1877; with medication data *n* = 1486)

^b^ Adjusted for age, sex, education, martial status, employment status, perceived financial situation, smoking status, current volume of ethanol consumed per year, CAGE score

## Discussion

In this cross-sectional survey of the general population in the Russian cities of Arkhangelsk and Novosibirsk the age-adjusted point prevalence of moderate depression (PHQ-9 ≥ 10) and anxiety (GAD-7 ≥ 10) varied between the two cities with a higher prevalence of both in Novosibirsk (moderate depression 10.6%; moderate anxiety 6.0%) than Arkhangelsk (moderate depression 6.3%; moderate anxiety 3.8%). In both cities the prevalence of common mental disorders varied by gender with higher prevalence in women. Reporting of ever having received a diagnosis of depression was 3 times lower than point prevalence for depression in men and 2 times lower in women. Use of anti-depressants and anxiolytic medications was extremely low with no men with symptoms of depression or anxiety receiving any pharmacological treatment (anti-depressants or anxiolytics), despite a higher use of health services and other medications among those with symptoms of depression and anxiety.

Separate studies of the prevalence of depression and anxiety have been carried out some years ago in both Arkhangelsk (the Arkhangelsk study 2000) and Novosibirsk (HAPIEE 1999–2000) which found substantially higher prevalences of both depression and anxiety. Russia has experienced rapid social and political change over time therefore it is feasible prevalence of common mental disorders may have changed in the time frame between these studies, however it is difficult to make formal comparisons between the three studies given differences in methodology for deriving case definitions. The more recent ESSE-RF survey from 2012 to 13 which included 10 regions in Russia used the HADS to assess depression symptoms also found higher prevalences of depression (25.6%) and anxiety (46.3%) closer to the earlier studies [12]. It is impossible to know from our data whether this indicates true variation or large differences based on assessment methods and/or recruitment of participants.

The prevalence of depression we have found is comparable to more recent population-based surveys in other countries which have used PHQ-9 to assess depression with estimates of moderate depression (PHQ-9 ≥ 10) from Germany [28], Hong Kong [29], Korea [30], Sweden [31] and the USA [30] ranging from 4.2% (Hong Kong) to 10.8% (Sweden). A population study using GAD-7 ≥ 10 from Germany [32] found a prevalence of anxiety of 5.9% similar to the prevalences found here, while studies from Malaysia [33] and Sweden [31] using a lower cut point of ≥8 found prevalences of 8.2 and 14.7% respectively (corresponding age-standardised prevalences≥8 here were 9.0% Novosibirsk and 6.0% Arkhangelsk). It should be noted that other population-based studies using GAD-7 and PHQ-9 included a wider age range of participants than were included here which limits comparability with our study findings. Also we found in this study that there were differences between the two cities which were not explained by socio-economic circumstances or health behaviours of the included study participants. While in our study the prevalence of both depression and anxiety was higher in Novosibirsk than Arkhangelsk, in contrast to Russian state statistics which show rates of mortality from suicide are higher in Arkhangelsk. This may be an indicator that the study is affected by selection bias. However if these findings represent true differences they suggest there are other between city level factors besides levels of reported symptoms which explain the higher suicide rates in Arkhangelsk, for example differences in alcohol use. Mortality from accidental poisoning by alcohol from Russian State statistics is also substantially higher in Arkhangelsk than Novosibirsk indicating a higher prevalence of hazardous drinking.

The levels of ever receiving a diagnosis and treatment for depression and anxiety in our study population were very low. Striking findings were both lower prevalence of reported lifetime diagnosis compared to point prevalence and the extremely low levels of use of anti-depressant and anxiolytic medications. Only 10 participants in the entire sample reported use of anti-depressants and 8 anxiolytics. No men with PHQ-9 ≥ 10 received any pharmacological treatment. This is consistent with a study of 155 primary care users in St Petersburg where none of the 55 participants identified as having symptoms of depression were receiving pharmacological treatment [13]. In contrast the other populations included in the same study medication use ranged from 4% in Be’er Sheva to 38% in Seattle [13]. Our finding are also in contrast to larger population-based studies in other populations such as the NHANES survey from the USA (2005–8) where among the general population 27.0% of those with moderate depression (PHQ-9 10–14) and 31.8% with major depression (≥15) received pharmacological treatment [34] and the 2014 Adult Psychiatric Morbidity Survey for England where 55% of those with depression according to the Clinical Interview Schedule - Revised (CIS-R) had received medication [35]. Findings are particularly striking given that anxiolytics (although not anti-depressants) are available without prescription in Russia therefore access is easier than in many other settings. However it is possible participants did not report medications used for these purpose either due to associated stigma or because they did not consider drugs obtained over the counter for self-management of mood counted as “medications” in the traditional sense. Here we considered only medications within the ATC classes and have not looked at self-medication with alternative medicinal treatments marketed for similar purposes.

Our findings in relation to ever having received a diagnosis are also substantially lower than the English study where 70% of participants with symptoms of depression had also received a diagnosis of depression at some stage in their life [35]. No data were available on use of non-pharmacological treatments (such as psychotherapy) in the current study. However given the low levels of participants reporting ever having received a diagnosis of depression or anxiety (in particular depression) it seems unlikely that the use of talking therapies over pharmacological management is the primary reason for the very low levels of pharmacological treatment found in this population. It seems more likely that we have identified here a very large gap in diagnosis and treatment of common mental disorders. This cannot be explained in terms of lack of contact with health services per se as even after restricting to those who did not report co-morbid physical illnesses and adjusting for socio-demographic and behavioural risk factors those reporting symptoms of depression and anxiety had more contact with health services and were taking more medications than those who did not. There was also a clear dose response with severity of symptoms. This is consistent with findings in other settings that use of (non-mental) health services is higher among those with mental health symptoms [20–22]. We have identified here missed opportunities for diagnosis and providing appropriate treatment for those with common mental health disorders. This may be influenced both by beliefs of doctors, but also by the attribution and presentation of symptoms by patients seeking help [36]. Low availability and perceived value of including mental health treatment within primary care may also be an important factor in explaining the treatment gap as currently the main options to receive treatment for mental health problems are through specialist services or private treatment, which is not affordable for the majority. In the study of primary care users in St Petersburg cost of treatment was raised by participants as a major barrier to treatment [37].

Our findings of low levels of treatment and diagnosis are consistent with previous studies on attitudes and beliefs about depression where presented with vignettes describing people with symptoms of depression Russian participants compared to participants from Germany [38] and the United States [39] were less likely to attribute depression to biological rather than psycho-social causes [38, 39], more likely to indicate depression was related to “weak will” [38, 39] and less likely to endorse help seeking for the individual [39], while in a similar comparative study Russian participants were less likely to endorse seeking help from medical sources than British participants and scored lower on a scales indicating tolerance towards descriptions of people with mental health problems [40]. Interventions aimed at improving mental health literacy [41–43] and reducing stigma [44, 45] both in the general population and among health care professionals could be of benefit in closing the treatment gap.

This study has several limitations to consider in interpretation of the findings:

Firstly all data on common mental disorders, including history of diagnosis and treatment, and health service use are self-reported. We were able to look at prevalence of depression and anxiety symptoms at one point in time based on symptoms in the past 2 weeks but this cannot be considered equivalent to clinical diagnosis and does not reflect longer term duration of symptoms. Although the PHQ-9 and GAD-7 are validated instruments they have not been validated for use in Russia. Interviews in this study were conducted face to face and it is possible that some participants may not have reported having received a diagnosis of depression or anxiety or disclosed medication use at the health check due to concerns about associated stigma. Furthermore the proportion of participants who brought their medications to the health check examination was small (27%). For the majority reporting was verbal only and some level of measurement error due to errors in reporting by participants who may not remember or be aware of the names of all their medication is also feasible. The low use of medications is however consistent with the very low reporting of diagnosis also found in this study.

Secondly this was a cross-sectional study and we are not able to determine the temporal direction of association between common mental disorder and health service use, particularly given the time frame for asking about symptoms of depression was the past 2 weeks and visits to doctors and hospital admissions the past 12 months. However given symptoms of common mental disorders are fairly persistent it is likely this does reflect pattern of symptoms in the past 12 months. Although analyses were restricted to those who did not report co-morbid physical health problems this was not an exhaustive list and there is still potential confounding due to co-morbidities not included or not reported by participants or undiagnosed at the time of the interview. However our findings were robust to stratification by the stated co-morbidities and controlling for a range of potential confounders.

Thirdly response rates for one city (Novosibirsk) were particularly low which may have affected the representativeness of the sample. Response rates for Arkhangelsk were higher but not 100% and it is possible that selection into the study may have been differential by mental health status. The impact of bias is difficult to estimate. Comparisons of the educational profile of the study participants with the 2010 census [23] showed ratio of the observed to expected was 0.98 (95% CI 0.92, 1.04) for Arkhangelsk but with variation over age with higher than expected education at younger ages and lower than expected education in the older participants. For Novosibirsk there was some evidence that educational profile of participants was higher than expected (1.14 (95% CI 1.07, 1.21)) but this was consistent across age groups. Those with more severe depression at baseline were less likely to attend the subsequent health check [23]. If the same were true for initial participation we may have under-estimated the prevalence of common mental disorders in this population. Within the two cities selection was within four districts. These were selected to represent a range of socio-demographic and mortality levels in each city but recruitment was not from all city districts so may not be generalisable to the whole city. To this extent prevalence estimates should be interpreted with caution however the lack of treatment in those identified here with moderate depression is valid and an important finding.

Finally our findings are not generalisable to the whole of Russia. Within the two cities included there was variation in point prevalence of depression and anxiety symptoms although the finding of strikingly low use of anti-depressants was consistent in both.

## Conclusions

In conclusion here we have identified a substantial gap in diagnosis and treatment of common mental disorders among the general population in two Russian cities. Further work understanding the barriers to diagnosis and appropriate treatment in Russia is needed in order to design appropriate interventions to improve provision of care for common mental disorders in this population.

## Supplementary Information

**Additional file 1: Supplementary Figure 1.** Distribution of PHQ-9 Severity Scores.

**Additional file 2: Supplementary Figure 2.** Distribution of GAD-7 Severity Scores.

**Additional file 3: Supplementary Figure 3.** Distribution of Number of visits to a doctor in the past 12 months.

**Additional file 4: Supplementary Figure 4.** Distribution of Number of Hospital visits in the past 12 months.

**Additional file 5: Supplementary Figure 5.** Distribution of the Number of Medications used.

**Additional file 6: Supplementary Table 1.** Distribution of Socio-demographic factors and health behaviours in Arkhangelsk and Novosibirsk.

**Additional file 7. Supplementary Table 2.** Associations between severity of depression and anxiety with use of health services and medications among those who report 1 or more physical health problems

## Data Availability

The data that support the findings of this study are available from Know Your Heart, but restrictions apply to the availability of these data and so are not publicly available. Data are available upon reasonable request and with permission of Know Your Heart Study (https://metadata.knowyourheart.science).

## Abbreviations

ATC code: Anatomical Therapeutical Chemical Code
CES-D: Centre for Epidemiologic studies depression scale
CIS-R: Clinical Interview Schedule - Revised
ESSE-RF: Epidemiology of Cardiovascular Diseases in various regions of Russia representative Federation
GAD-7: Generalised Anxiety Disorder Assessment 7
HADS: Hospital Anxiety and Depression Scale
HAPIEE: Health, Alcohol and Psychosocial factors in Eastern Europe
IQR: Interquartile range
NHANES: National Health and Nutrition Examination Survey
PHQ-9: Patient Health Questionnaire 9
USA: United States of America
WHO: World Health Organisation
