# Supplementary material for: Prevalence of symptoms, ever having received a diagnosis and treatment of depression and anxiety, and associations with health service use amongst the general population in two Russian cities
Source: BMC Psychiatry. 2020 Nov 12;20:537. doi: 10.1186/s12888-020-02938-w (PMC7663865; doi:10.1186/s12888-020-02938-w)
Supplement: Supplementary file 6 — Additional file 6: Supplementary Table 1. Distribution of Socio-demographic factors and health behaviours in Arkhangelsk and Novosibirsk. [file 12888_2020_2938_MOESM6_ESM.docx]

**Supplementary Table 1. Distribution of Socio-demographic factors and health behaviours in Arkhangelsk and Novosibirsk**

|  | | Novosibirsk | | Arkhangelsk | |
| --- | --- | --- | --- | --- | --- |
|  |  | N | (%) | N | (%) |
| Age | 35-39 | 231 | (8.8) | 239 | (9.8) |
|  | 40-44 | 293 | (11.2) | 357 | (14.6) |
|  | 45-49 | 352 | (13.4) | 338 | (13.8) |
|  | 50-54 | 365 | (13.9) | 369 | (15.1) |
|  | 55-59 | 385 | (14.7) | 381 | (15.5) |
|  | 60-64 | 482 | (18.4) | 375 | (15.3) |
|  | 65-69 | 517 | (19.7) | 393 | (16.0) |
| Sex | Male | 1151 | (43.9) | 1022 | (41.7) |
|  | Female | 1474 | (56.2) | 1430 | (58.3) |
| Education | Lower than secondary | 194 | (7.4) | 183 | (7.5) |
|  | Secondary | 1346 | (51.3) | 1330 | (54.2) |
|  | Tertiary | 1085 | (41.3) | 939 | (38.3) |
| Perceived financial situation | Not enough for food | 76 | (3.0) | 60 | (2.5) |
|  | Enough for food but not clothes | 567 | (22.4) | 320 | (13.1) |
|  | Enough for food and clothes but difficult to buy large domestic appliances | 1303 | (51.4) | 1184 | (48.5) |
|  | Enough for large domestic appliances but difficult to buy a new car | 514 | (20.3) | 687 | (28.2) |
|  | Enough for a large car but difficult to buy a flat or house | 48 | (1.9) | 121 | (5.0) |
|  | No financial constraints | 29 | (1.1) | 68 | (2.8) |
|  | Missing | 88 |  | 12 |  |
| Employment status | In regular paid employment | 1441 | (55.0) | 1544 | (63.0) |
|  | Not in regular paid employment | 1181 | (45.0) | 908 | (37.0) |
|  | Missing | 3 |  | 0 |  |
| Smoking Status | Never Smoker | 1226 | (46.7) | 1286 | (52.5) |
|  | Ex-smoker | 619 | (23.6) | 559 | (22.8) |
|  | Current Smoker | 778 | (29.7) | 607 | (24.8) |
|  | Missing | 2 |  | 0 |  |
| Volume of ethanol | Non drinker | 640 | (24.5) | 426 | (17.4) |
|  | <2 Litres/year | 1141 | (43.7) | 1214 | (49.5) |
|  | 2-4.99 litres/year | 347 | (13.3) | 352 | (14.4) |
|  | 5-9.99 Litres/year | 258 | (9.9) | 232 | (9.5) |
|  | 10-19.99 Litres/year | 143 | (5.5) | 137 | (5.6) |
|  | >20 Litres/year | 84 | (3.2) | 90 | (3.7) |
|  | Missing | 12 |  | 1 |  |
| CAGE score | 0 | 2085 | (79.4) | 1876 | (76.5) |
|  | 1 | 247 | (9.4) | 239 | (9.8) |
|  | 2 | 142 | (5.4) | 188 | (7.7) |
|  | 3-4 | 151 | (5.8) | 149 | (6.1) |
| Total | | 2625 | (100) | 2452 | (100) |
