# Supplementary material for: Prevalence of symptoms, ever having received a diagnosis and treatment of depression and anxiety, and associations with health service use amongst the general population in two Russian cities
Source: BMC Psychiatry. 2020 Nov 12;20:537. doi: 10.1186/s12888-020-02938-w (PMC7663865; doi:10.1186/s12888-020-02938-w)
Supplement: Supplementary file 7 — Additional file 7. Supplementary Table 2. Associations between severity of depression and anxiety with use of health services and medications among those who report 1 or more physical health problems [file 12888_2020_2938_MOESM7_ESM.docx]

**Supplementary Table 2. Associations between severity of depression and anxiety with use of health services and medications among those who report 1 or more physical health problems**

|  | Number of visits to doctor in past 12 months  (missing=6) | | | | Number of hospital visits in past 12 months | | | | Number of medications used | | | |
| --- | --- | --- | --- | --- | --- | --- | --- | --- | --- | --- | --- | --- |
| PHQ-9 Severity score | Median  (IQR) | Median  (IQR)^a^ | Age, sex and city adjusted rate ratio^a^ | Fully adjusted rate ratio^a^,^b^ | Median  (IQR) | Median  (IQR)^a^ | Age, sex and city adjusted rate ratio^a^ | Fully adjusted rate ratio^a,b^ | Median  (IQR) | Median  (IQR)^a^ | Age, sex and city adjusted rate ratio^a^ | Fully adjusted rate ratio^a,b^ |
| No symptom (<5) | 2 (0-4) | 2 (0-6) | 1.00 (ref) | 1.00 (ref) | 0 (0-0) | 0 (0-0) | 1.00 (ref) | 1.00 (ref) | 1 (0-2) | 1 (0-3) | 1.00 (ref) | 1.00 (ref) |
| Mild depression  (5-9) | 3 (1-7) | 4 (1-8) | 1.38 (1.27, 1.50) | 1.37 (1.25, 1.49) | 0 (0-0) | 0 (0-0) | 1.34 (1.11, 1.62) | 1.30 (1.08, 1.58) | 1 (0-3) | 2 (0-3) | 1.26 (1.15, 1.37) | 1.25 (1.14, 1.36) |
| Moderate depression  (10-14) | 4 (1-8) | 5 (2-8) | 1.50 (1.31, 1.73) | 1.47 (1.28, 1.69) | 0 (0-1) | 0 (0-1) | 1.76 (1.32, 2.36) | 1.62 (1.21, 2.16) | 2 (0-3.25) | 2 (0-4) | 1.40 (1.22, 1.61) | 1.34 (1.17, 1.54) |
| Major depression (>15) | 5 (1-10) | 5 (1-10) | 1.65 (1.35, 2.00) | 1.54 (1.27, 1.88) | 0 (0-1) | 0 (0-1) | 2.06 (1.41, 3.01) | 1.72 (1.18, 2.53) | 2 (1-3) | 2 (1-3) | 1.41 (1.15, 1.74) | 1.30 (1.06, 1.60) |
| Test for trend |  |  | P<0.001 | P<0.001 |  |  | P<0.001 | P<0.001 |  |  | P<0.001 | P<0.001 |
| GAD-7 Anxiety severity score |  |  |  |  |  |  |  |  |  |  |  |  |
| No symptoms (<5) | 2 (0-5) | 3 (1-6) | 1.00 (ref) | 1.00 (ref) | 0 (0-0) | 0 (0-0) | 1.00 (ref) | 1.00 (ref) | 1 (0-2) | 1 (0-3) | 1.00 (ref) | 1.00 (ref) |
| Mild anxiety (5-9) | 3 (1-7) | 4 (1-8) | 1.29 (1.17, 1.43) | 1.27 (1.15, 1.40) | 0 (0-0) | 0 (0-0) | 1.32 (1.07, 1.62) | 1.29 (1.05, 1.59) | 1 (0-3) | 1.5 (0-3) | 1.08 (9.98, 1.19) | 1.06 (0.96, 1.17) |
| Moderate anxiety (10-14) | 3 (0-8) | 4 (1-8) | 1.34 (1.10, 1.62) | 1.28 (1.06, 1.55) | 0 (0-0) | 0 (0-0) | 1.14 (0.74, 1.76) | 1.03 (0.67, 1.58) | 1 (0-3) | 2 (0-3) | 1.19 (0.97, 1.45) | 1.15 (0.94, 1.40) |
| Severe anxiety (>15) | 4 (1-8) | 5 (2-9) | 1.43 (1.09, 1.86) | 1.40 (1.08, 1.83) | 0 (0-1) | 0 (0-1) | 2.32 (1.44, 3.74) | 2.04 (1.26, 3,28) | 2 (0-4) | 2 (1-4) | 1.20 (0.91, 1.57) | 1.15 (0.88, 1.50) |
| Test for trend |  |  | P<0.001 | P<0.001 |  |  | P<0.001 | P=0.002 |  |  | P=0.02 | P=0.06 |

^a^Restricted to those reporting a diagnosis of cancer, angina, stroke, rheumatoid arthritis, osteoarthritis, asthma, diabetes, chronic lung disease, myocardial infarction, kidney disease or heart failure and no missing data on covariates (n=3084; with medication data n=2,500)

^b^ Adjusted for age, sex, education, martial status, employment status, marital status, perceived financial situation, smoking status, current volume of ethanol consumed per year, CAGE score
