# Supplementary material for: Prevalence of symptoms, ever having received a diagnosis and treatment of depression and anxiety, and associations with health service use amongst the general population in two Russian cities
Source: BMC Psychiatry. 2020 Nov 12;20:537. doi: 10.1186/s12888-020-02938-w (PMC7663865; doi:10.1186/s12888-020-02938-w)
Supplement: Supplementary file 4 [file 12888_2020_2938_MOESM2_ESM.docx]

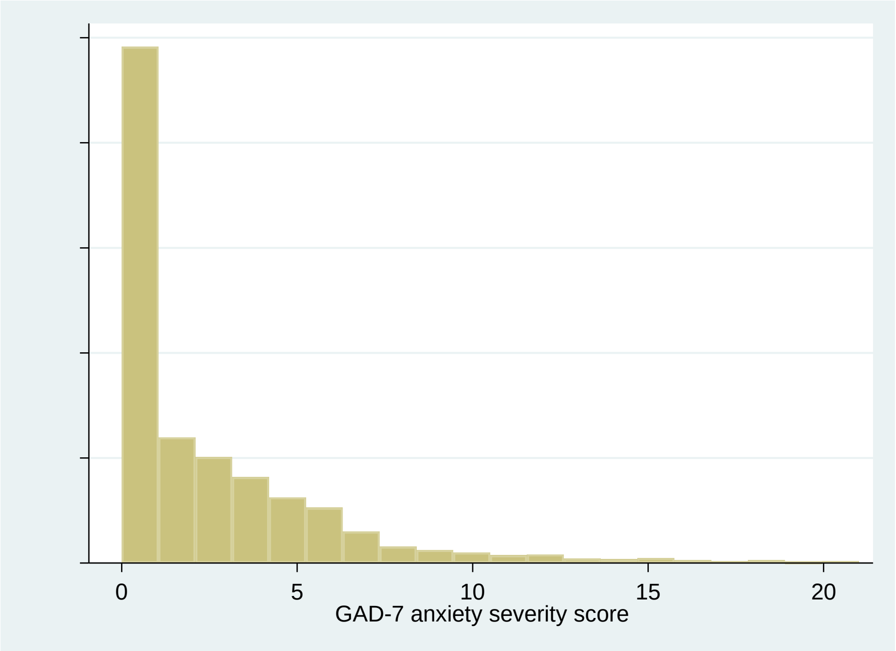
0
5
10
15
20
GAD-7 anxiety severity score
